# Supplementary figures and images for: Detrimental role of IL-33/ST2 pathway sustaining a chronic eosinophil-dependent Th2 inflammatory response, tissue damage and parasite burden during Toxocara canis infection in mice
Source: PLoS Negl Trop Dis. 2021 Jul 29;15(7):e0009639. doi: 10.1371/journal.pntd.0009639 (PMC8354467; doi:10.1371/journal.pntd.0009639)

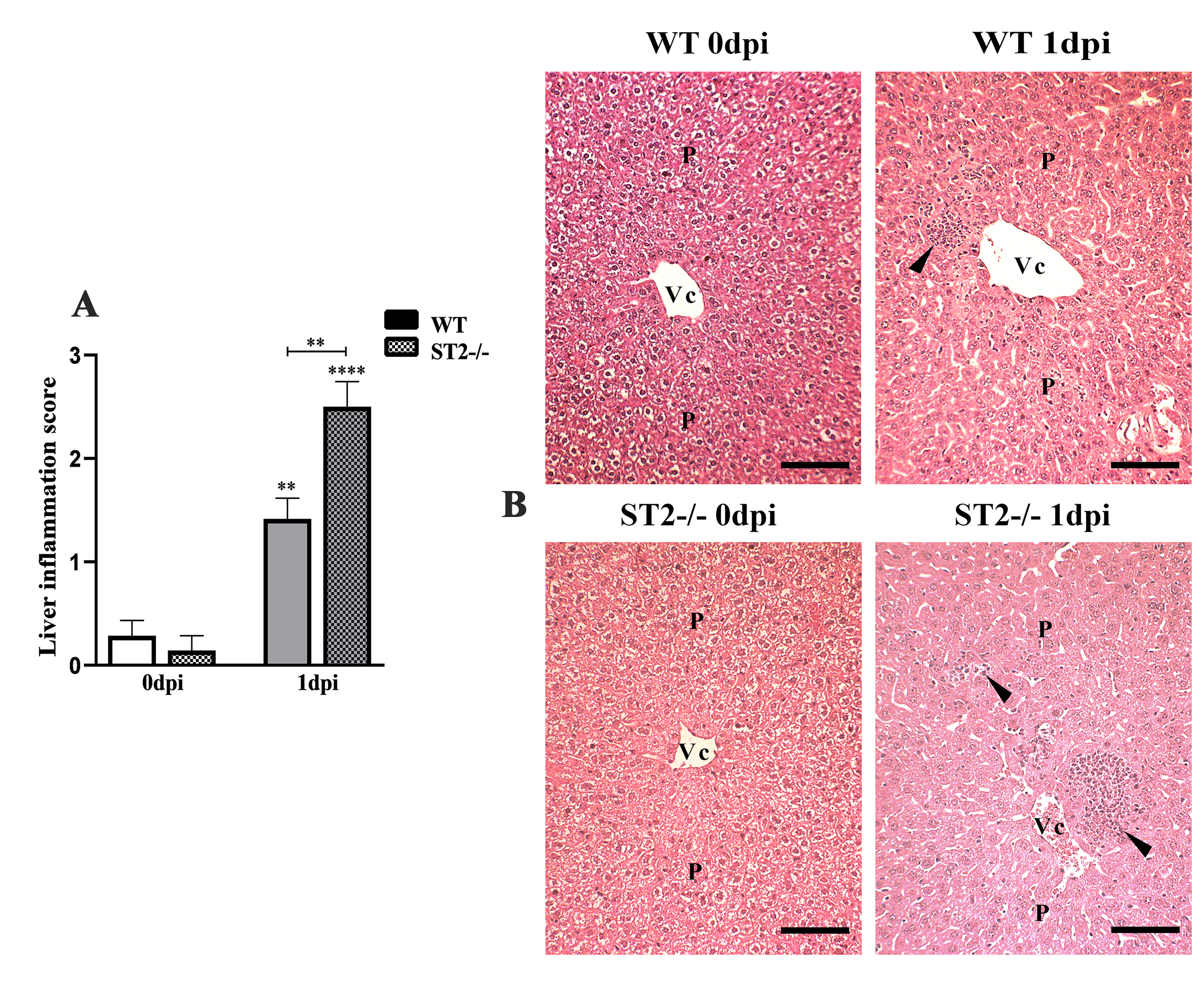

Supplement: S1 Fig — (A) Liver inflammation score; (B) Representative hematoxylin and eosin staining of liver sections, hepatic parenchyma (P), vein lobular center (Vc), inflammatory infiltration foci (arrowheads). Bar = 200μm. Statistical comparisons were made between each strain with its specific uninfected group (0dpi) represented by the asterisk without the bar and between strains at the same time of infection represented by the asterisk with the bar. Results represent mean ± S.E.M., **p<0.01, ****p<0.0001. One-way ANOVA test and Kruskal-Wallis test followed by Dunn’s test were used. (TIF) [file pntd.0009639.s001.tif]

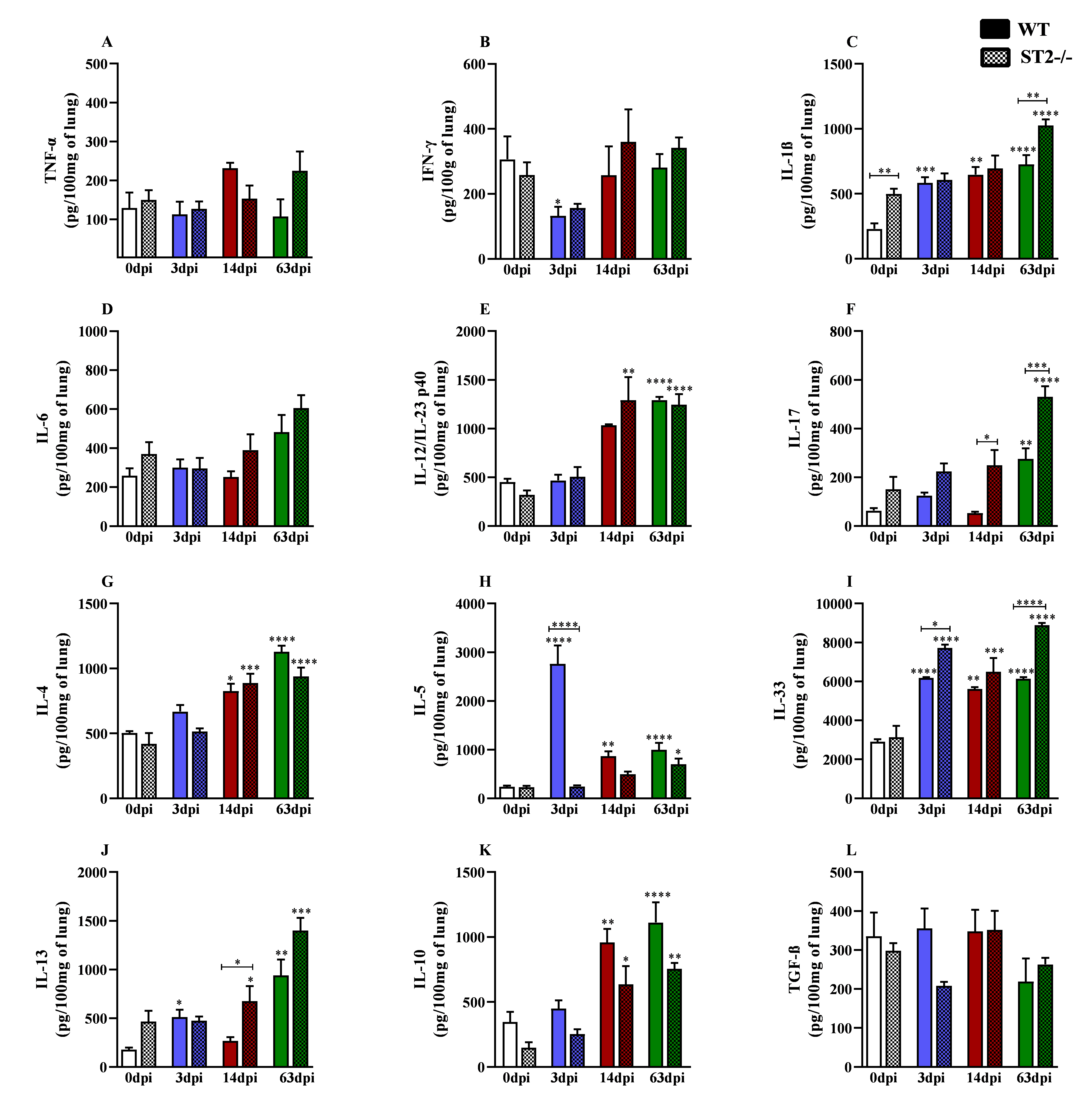

Supplement: S2 Fig — (A) TNF-α; (B) IFN-γ; (C) IL-1-ß; (D) IL-6; (E) IL-12/IL-23p40; (F) IL-17; (G) IL-4; (H) IL-5; (I) IL-33; (J) IL-13; (K) IL-10; (L) TGF-ß. Statistical comparisons were made between each strain with its specific uninfected group (0dpi) represented by the asterisk without the bar and between strains at the same time of infection represented by the asterisk with the bar. Results represent mean ± S.E.M., *p<0.05, **p<0.01, ***p<0.001, ****p<0.0001. One-way ANOVA test and Kruskal-Wallis test followed by Dunn’s test were used. (TIF) [file pntd.0009639.s002.tif]
