# Supplementary material for: Detrimental role of IL-33/ST2 pathway sustaining a chronic eosinophil-dependent Th2 inflammatory response, tissue damage and parasite burden during Toxocara canis infection in mice
Source: PLoS Negl Trop Dis. 2021 Jul 29;15(7):e0009639. doi: 10.1371/journal.pntd.0009639 (PMC8354467; doi:10.1371/journal.pntd.0009639)
Supplement: S1 Table — (PDF) [file pntd.0009639.s003.pdf]

S3 Table 1. Main findings in the liver, lung and brain in histopathological analysis in *T. canis* infection.

Group	Liver	Lung	Brain
**WT 3dpi**	Larvae dispersed in the parenchyma; necrotic areas; hemorrhage; vascular congestion; inflammatory foci mixed composed of eosinophils, neutrophils, macrophages and lymphocytes.	Interalveolar septal thickening with mixed inflammatory infiltrate; perivascular edema; hemorrhagic areas; larvae in the lung parenchyma; bronchial and bronchiolar cell hypertrophy; presence of granulomas in the exudative phase.	Multifocal areas of hemorrhage; vacuolation of the neuropil in the cerebral cortex; hemorrhagic foci in the cerebellum and hippocampus; Inflammatory infiltrates in the cerebral cortex.
**ST2$^{-/-}$ 3dpi**	Larvae dispersed in the parenchyma; necrotic areas; hemorrhage; vascular congestion; inflammatory foci mixed composed of neutrophils, macrophages and lymphocytes.	Interalveolar septal thickening with mixed inflammatory infiltrate; perivascular edema; hemorrhagic areas; larvae in the lung parenchyma; bronchial and bronchiolar cell hypertrophy.	Multifocal areas of hemorrhage; vacuolation of the neuropil in the cerebral cortex; hemorrhagic foci in the cerebellum and hippocampus; inflammatory infiltrates in the cerebral cortex.
**WT 14dpi**	Inflammatory foci in the parenchyma; presence of granulomas in the exudative phase.	Macrophages with brownish pigment in the cytosol; presence of granuloma in the exudative phase.	Hemorrhagic foci in the cerebrum.
**ST2$^{-/-}$ 14dpi**	Inflammatory foci in the parenchyma with few eosinophils and lymphocytes; presence of few granulomas in the exudative phase.	Macrophages with brownish pigment in the cytosol; diffuse inflammatory infiltrate with little presence of neutrophils and eosinophils.	Hemorrhagic foci in the cerebrum.
**WT 63dpi**	Inflammatory foci in the hepatic and perivascular parenchyma; vascular congestion; presence of granulomas in the exudative and productive phase.	Thickening of the interalveolar septa; diffuse inflammatory infiltrate with formation of BALT; small hemorrhagic foci; capillary congestion; presence of granulomas in the production phase.	Focal areas with “gitter cells” in the white matter of the cerebellar folia; vacuolization areas and gliosis foci; reactive blood vessels in the cerebellum and cerebrum; presence of larvae and hemorrhagic foci in the cerebrum and hippocampus; perivascular accumulation of hemosiderophages in the brain.
**ST2$^{-/-}$ 63dpi**	Inflammatory foci in the hepatic and perivascular parenchyma with few eosinophils; vascular congestion; presence of few granulomas in the exudative and productive phase.	Thickening of the interalveolar septa; diffuse inflammatory infiltrate with formation of BALT; small hemorrhagic foci; capillary congestion; presence of few granulomas.	Focal areas with “gitter cells” in the white matter of the cerebellar folia; vacuolization areas and gliosis foci; reactive blood vessels in the cerebellum and cerebrum; few hemorrhagic foci; perivascular accumulation of hemosiderophages in the brain.
