# Supplementary material for: Detrimental role of IL-33/ST2 pathway sustaining a chronic eosinophil-dependent Th2 inflammatory response, tissue damage and parasite burden during Toxocara canis infection in mice
Source: PLoS Negl Trop Dis. 2021 Jul 29;15(7):e0009639. doi: 10.1371/journal.pntd.0009639 (PMC8354467; doi:10.1371/journal.pntd.0009639)
Supplement: Attachment — Submitted filename: PLOS NTD Comments.docx [file pntd.0009639.s004.docx]

**Comments**

- Authors of the manuscript report that IL-33/ST2 pathway sustained the Th2 immune

response contributing to eosinophil activity, tissue damage and parasite tropism during infection by *T. canis* in mice. In this study authors have mechanistically also proven their hypothesis. Reported results are very interesting and novel.

- Authors have shown the Interleukin-33 (IL-33) related IL-1 family cytokines in the table format, for the better understanding it can be shown in graphical format.
- It will be also good if authors could perform one or two cellular based experiments using the flow cytometry, it will further strengthen their current findings.
