# Supplementary material for: Detrimental role of IL-33/ST2 pathway sustaining a chronic eosinophil-dependent Th2 inflammatory response, tissue damage and parasite burden during Toxocara canis infection in mice
Source: PLoS Negl Trop Dis. 2021 Jul 29;15(7):e0009639. doi: 10.1371/journal.pntd.0009639 (PMC8354467; doi:10.1371/journal.pntd.0009639)
Supplement: Attachment — Submitted filename: Rebuttal letter Manuscript Leal-Silva et al 2021 Plos Neg.docx [file pntd.0009639.s005.docx]

**Manuscript "Detrimental role of IL-33/ST2 pathway sustaining a chronic eosinophil-dependent Th2 inflammatory response, tissue damage and parasite burden during *Toxocara canis* infection in mice"**

**Summary and General Comments**

**Reviewer #1: This work from Leal-Silva et al. is a comprehensive comparison of the effect of a controlled T. canis infection in wildtype (WT)and ST2-knock-out (KO) mice. The ‘n’ values are of a good size (n=6-7), although I am a bit surprised by how tight some of the data are in the murine immune assays. This is a nice description of the impact of a global KO of the ability for IL-33 to signal through ST2. The work makes a contribution to the field. Attention to the following issues is needed**

1) **A major concern is that the WT mice and KO mice will have different gut microbiotas and data are not omnipresent on how the gut bacteria effects host physiology/immunology (e.g. a single species difference – segmented filamentous bacteria – was critical to Th17 immunity). The advised way to do the experiments is to cross WT with KO, get the heterozygotes, cross them and then use WT and KO from the same litters. It is not reasonable to ask the authors to do this to repeat their study (although some comparison of KO mice (from San Paulo) with KO mice bred in this way would be advantageous). It is critical that the authors acknowledge this difference and state it clearly in the discussion as a possibility that could explain some of the differences between the two strains.**

We appreciate your consideration regarding the importance of intestinal microbiota in our study, so we inserted in the discussion of our manuscript that the difference in microbiota between strains may interfere with the immune response, suggesting its role in controlling larval migration (lines 600-608).

We also added the following references:

Brint EK, Xu D, Liu H, Dunne A, McKenzie ANJ, O’Neill LAJ, et al. ST2 is an inhibitor of interleukin 1 receptor and Toll-like receptor 4 signaling and maintains endotoxin tolerance. Nat Immunol. 2004;5: 373–379. doi:10.1038/ni1050

Ivanov II, Littman DR. Segmented filamentous bacteria take the stage. Mucosal Immunol. 2010;3: 209–212. doi:10.1038/mi.2010.3

2) **The discussion is too long (8 pages) and is mostly an expanded version of the descriptive results. This needs to be shortened and focused to highlight the point of this paper. If I get it right, that is, in this mouse model IL-33 signaling through ST2 exaggerates immunopathology to *T. canis.***

The discussion was shortened to 6 pages, focusing on the immunopathology of the IL-33/ST2 pathway in *T. canis* infection.

3) **While noting the lengthy discussion, it would be of value to the reader for the authors to highlight that this is global ST2-KO, and then present some target cells in which absence of ST2 might play critical roles in the biology of *T. canis* infection.**

As suggested, a paragraph was added (lines 569-572) regarding the effects of the total absence of the ST2 receptor on immune cells and its possible effects on *T. canis* infection.

4) **With such a lot of descriptive data, perhaps a summary ‘carton’ figure would help the reader integrate and conceptualize the study.**

A summary ‘carton’ figure has been included as Figure 9.

5) **Are all the data for a single experiment with 13 mice per time-point? If so, please state this clearly in the methods or results section. Why are only female mice used?**

Yes, all data are from a single experiment with 13 animals, we added this information in the methodology (lines 169-170). Studies have shown that there is no difference in parasite load between males and females. The females were chosen because they had already been used in a previous experiment in our research group, as shown in this study:

Resende NMNM, Gazzinelli-Guimarães PH, Barbosa FS, Oliveira LM, Nogueira DS, Gazzinelli-Guimarães AC, et al. New insights into the immunopathology of early Toxocara canis infection in mice. Parasit Vectors. 2015;8: 354. doi:10.1186/s13071-015-0962-7

6) **Methods: were all the analysis blinded? This would be particularly important in the histological analysis.**

The parasite load, cytokines and histopathological analysis were performed blindly. The information regarding the histopathological analysis was added in the methodology (lines 284-285).

7) **Fig. 1: there appears to be a baseline (day 0) increase in leucocytes in ST-KO. This is worth commentary.**

We add this result to the text (lines 351-353).

8) **Fig. 2. If the * in panels a and b indicate a difference between WT and KO mice, I would be skeptical of these finding and doubt any biological relevance. The data points literally overlap.**

In Figure 2, * represents the statistical difference between the WT and KO mice, the letter "a" represents the difference between uninfected WT and infected WT, and the letter "b" represents the difference between uninfected KO and infected KO. We really cannot assert that the differences in antibody concentration between the WT and KO groups, while significantly statistical, could have biological relevance. Thus, a new discussion was carried out, emphasizing the need for further studies between the IL-33/ST2 pathway and the humoral response in *T. canis* infection (lines 588-595).

**Reviewer #2: The manuscript by Thaís Leal-Silva et al., is an important paper that not only confirms other studies showing e IL-33/ST2 pathway is involved in eosinophilia, hepatic and cerebral parasitic burden and induces the formation of granulomas related to tissue damage and pulmonary dysfunction, but advances the field by looking into mechanisms that might explain the effects of helminths. The work is of very high quality and has carefully analyzed and reported the data. However, I have few concerns.**

1. **Authors could include spaghetti plot giving better details for each subject at different time points**

Spaghetti plot graphs were included in the data on the number of larvae recovered from liver (Figure 3A), lung (Figure 5A) and brain (Figure 8A).

2. **Authors could check for the legends and patterns in the graphs**

The legends and patterns on the graphics were checked.

**3. Authors could show the relationship between parasitic burden, antibody levels, leukocyte profiles and the cytokines levels to strengthen their hypothesis**

We performed the correlation test between several variables, liver and brain parasitic load with peripheral blood eosinophils, liver and brain parasitic load with cytokines and no statistically significant results were observed, so we thought it better not to include them in the research.

**4. Authors could include table for the comparison of histopathology in liver, lungs and brain from Wild-Type (WT) BALB/c**

We have added as supplementary figure 3 the table with the main histopathological findings in liver, lung and brain.

**Reviewer #3: Comments**

**Authors of the manuscript report that IL-33/ST2 pathway sustained the Th2 immune response contributing to eosinophil activity, tissue damage and parasite tropism during infection by *T. canis* in mice. In this study authors have mechanistically also proven their hypothesis. Reported results are very interesting and novel.**

1. **Authors have shown the Interleukin-33 (IL-33) related IL-1 family cytokines in the table format, for the better understanding it can be shown in graphical format.**

We added as Supplementary Figure 2 the cytokine data in bar graph format.

2. **It will be also good if authors could perform one or two cellular based experiments using the flow cytometry, it will further strengthen their current findings.**

We also believe that the use of the flow cytometry technique would enrich our research and broaden our understanding of cells acting during *T. canis* infection; however, due to the coronavirus pandemic, brazilian government research funding cuts and restriction measures of people in brazilian universities in order to increase social isolation, prevent us from expanding this research and performing this technique at this time. However, in the near future, we are planning to carry out studies using flow cytometry to verify the cell type acting in *T. canis* infection and the cytokines expressed by these cells.
